# On the convergence rate of the boosted difference-of-convex algorithm (DCA)

**DOI:** 10.1007/s11590-026-02305-w

**Published:** 2026-05-19

**Authors:** Hadi Abbaszadehpeivasti, Etienne de Klerk, Adrien Taylor

**Affiliations:** 1 Tilburg University, Tilburg, Netherlands; 2 INRIA, Paris, France

**Keywords:** Boosted DCA, Semidefinite programming, Performance estimation, Difference of convex functions

## Abstract

The difference-of-convex algorithm (DCA) is a well-established nonlinear programming technique that solves successive convex optimization problems. These sub-problems are obtained from the difference-of-convex (DC) decompositions of the objective and constraint functions. We investigate the worst-case performance of the unconstrained DCA, with and without boosting, where boosting simply performs an additional step in the direction generated by the usual DCA method. We show that, for certain classes of DC decompositions, the boosted DCA is provably better in the worst-case than the usual DCA. While several numerical studies have reported that boosted DCA outperforms classical DCA, a theoretical explanation for this behavior has, to the best of our knowledge, not been given until now. Our proof technique relies on semidefinite programming (SDP) performance estimation.

## Introduction

We consider the unconstrained difference-of-convex (DC) optimization problem

1 $$\begin{aligned} f^\star :=&\inf f(x)\nonumber \\&\, \text {s.t.} \ x\in {\mathbb {R}}^n \end{aligned}$$

where $$f: {\mathbb {R}}^n\rightarrow {\mathbb {R}}$$ is a *difference-of-convex (DC)* function, i.e. $$ f=f_1-f_2, $$ where $$f_1$$ and $$f_2$$ are closed convex proper functions. We also assume that the infimum $$f^\star $$ is finite, but not necessarily attained. We will further restrict ourselves to the case where $$f_1$$ and $$f_2$$ are so-called functions of bounded curvature. To this end, recall that a function *f* has a *maximum curvature* $$0 \le L < \infty $$ if $$ x \mapsto \tfrac{L}{2}\Vert x\Vert ^2-f(x)$$ is convex, and *minimum curvature* $$\mu > -\infty $$ if $$ x \mapsto f(x)-\tfrac{\mu }{2}\Vert x\Vert ^2$$ is convex. The class of functions with minimum curvature *μ * and maximum curvature *L* will be denoted by $${\mathcal {F}}_{\mu ,L}({\mathbb {R}}^n)$$.

The celebrated difference-of-convex (DCA) algorithm for problem (1) may be stated as follows.

**Algorithm 1:**
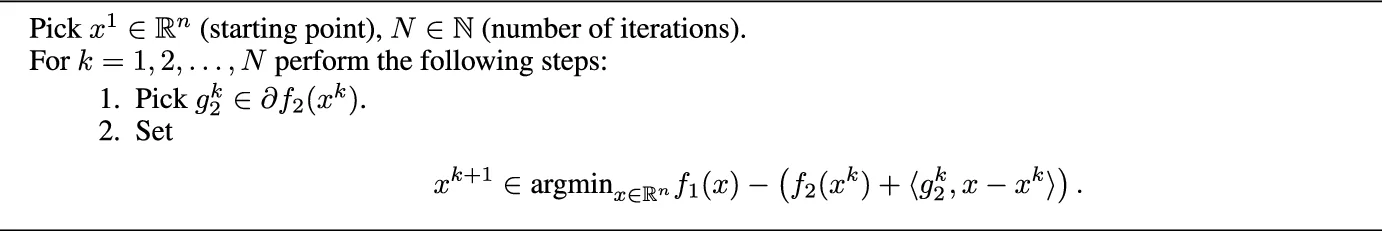
DCA

In the statement of Algorithm 1, $\partial f_i(x^k)$ (*i=1,2*) refers to the set of sub-gradients of $f_i$ at $x^k$. In this paper, we will only present new results for the case where $f_1$ and $f_2$ both have finite maximum curvature, which implies that both functions are differentiable and *L*-smooth. However, we will develop the framework of our analysis in the general setting, and therefore will refer to sub-gradients when we do not assume smoothness.

The first convergence results for Algorithm 1 are attributed to [18, Theorem 3(iv)], namely if the sequence of iterates $\{x^k\}$ is bounded, then each accumulation point $x^\star$ of this sequence is a critical point of problem (1), i.e. $\partial f_1(x^\star )\cap \partial f_2(x^\star ) \ne \emptyset$. For the history and properties of the DCA we refer to the surveys by Le Thi and Dinh [12, 13] and Lipp and Boyd [14].

A more recent variant of the DCA, the so-called boosted DCA [3, 4, 9], extends the DCA step as follows.

**Algorithm 2:**
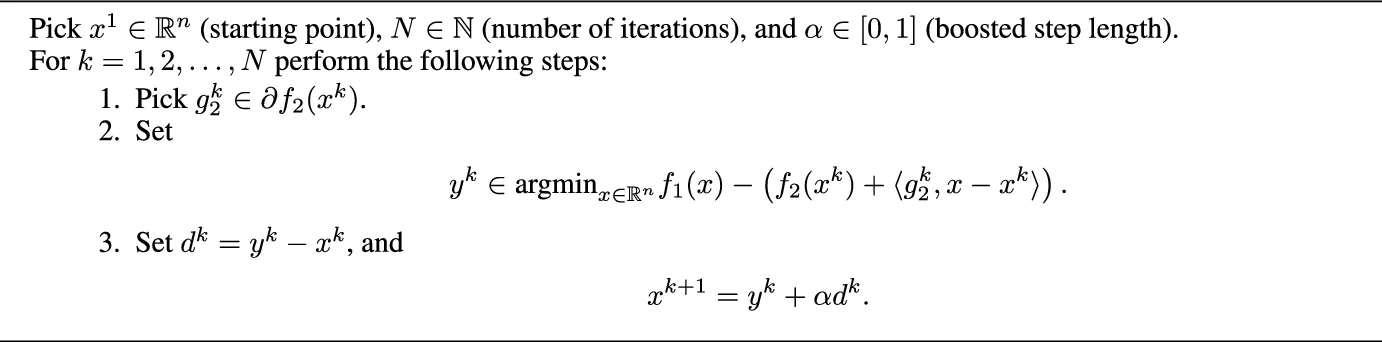
Boosted DCA

Note that the case *α = 0* corresponds to the usual DCA. In both Algorithms 1 and 2 we assume that a subgradient of $f_2$ is available at each iteration. The additional computational burden per iteration for boosted DCA is usually negligible compared to that of solving the intermediary subproblems and that of computing subgradients. It is therefore natural to compare the worst-case performance of the two methods on the same number of iterations.

The boosted DCA is motivated by the following lemma.

### Lemma 1

(Proposition 4 in [3]) Assume that *f* is continuously differentiable, and $f_1$ and $f_2$ have minimum curvature *μ > 0*. Then, with reference to Algorithm 2, one has

$$\begin{aligned} \langle \nabla f(y^k), d^k\rangle \le - \mu \Vert d^k\Vert ^2. \end{aligned}$$

In other words, if *f* is smooth, and $f_1,f_2$ strongly convex, it is possible to reduce *f* further along the DCA direction $d^k$, i.e. $d^k$ is a descent direction at $y^k$. This work investigates how different performance measures depend on the choice of “boosted" step length *α *. Our goal is to give a theoretical explanation of the advantage that one may obtain using boosted DCA as opposed to DCA. This, in turn, is motivated by successful numerical experiments in [3, 22], which show that boosted DCA typically outperforms DCA in practice.

In the literature, BDCA often involves some sort of line search. We will primarily deal with the case that the boosted step length *α * is a fixed constant, but we will also show how our analysis may be adapted to deal with some backtracking line search variants.

### Contributions of this paper

This work shows that boosted DCA enjoys the following worst-case convergence guarantee.

#### Theorem 1

Let $f_1\in {\mathcal {F}}_{\mu , L}({{\mathbb {R}}^n})$ and $f_2\in {\mathcal {F}}_{\mu , L}({{\mathbb {R}}^n})$, $0 \le \mu< L < \infty$. After *N* iterations of boosted DCA (Algorithm 2), one has,

$$\begin{aligned} { \min _{1\le k\le N+1}\frac{\left\| \nabla f(x^k)\right\| ^2}{L} \equiv } \min _{1\le k\le N+1}\frac{\left\| g_1^k-g_2^k\right\| ^2}{L}\le \frac{(f(x^1)-f^\star ) }{(1+\kappa \alpha )N + \frac{1}{2(1-\kappa )}}, \end{aligned}$$

where $\kappa := \mu /L \in [0,1)$, and where $0 \le \alpha \le \min \{1,2\kappa \}$.

When *α = 0*, this result reduces to a known (tight) convergence result for DCA, namely a special case of Theorem 3.1 in [1]. As soon as *κ > 0*, Theorem 1 shows that improved performance guarantees can be obtained with *α > 0*. On the other hand, when *μ = 0*, one has *κ = 0*, and Theorem 1 does not offer any improvement for positive *α *. Thus, we only show improvement from the boosted step if $f_1$ and $f_2$ are strongly convex.

The bound in Theorem 1 can be tight, as the following example shows.

#### Example 1

We will construct a univariate, piecewise quadratic function where the bound in Theorem 1 holds with equality for the parameter choices:

$$\begin{aligned} \mu = 1, \; L = 2, \; \kappa = \frac{1}{2}, \; \alpha = 1, \text{ and } N = 1. \end{aligned}$$

To this end, define the points

$$\begin{aligned} x^1 = 0, \; y^1 = \frac{-1}{\sqrt{5}}, \; x^2 = \frac{-2}{\sqrt{5}}, \; x_\star =\frac{-4}{\sqrt{5}}. \end{aligned}$$

We will construct functions $f_1 \in {\mathcal {F}}_{\mu , L}({{\mathbb {R}}})$ and $f_2 \in {\mathcal {F}}_{\mu , L}({{\mathbb {R}}})$ such that $x_\star$ is the minimizer of $f = f_1 - f_2$ on ${{\mathbb {R}}}$, $f(x_\star ) = 0$, and the values $x^2$ and $y^1$ are generated by Algorithm 2 from the starting point $x^1$, where $f(x^1) = 1$. Define the piecewise quadratic function,

$$\begin{aligned} f_1(x):= {\left\{ \begin{array}{ll} \frac{1}{2}x^2 - \frac{1}{\sqrt{5}}x - \frac{12}{5}, & x\le x^\star ,\\ x^2 + \frac{3}{\sqrt{5}}x - \frac{4}{5}, & x^\star \le x\le x^{2},\\ \frac{1}{2}x^2 + \frac{1}{\sqrt{5}}x - \frac{6}{5}, & x^{2}\le x\le y^1,\ \ \\ x^2 + \frac{2}{\sqrt{5}}x - \frac{11}{10}, & y^1\le x,\ \ \end{array}\right. } \end{aligned}$$

and similarly let

$$\begin{aligned} f_2(x):= {\left\{ \begin{array}{ll} \frac{1}{2}x^2 - \frac{1}{\sqrt{5}}x - \frac{12}{5}, & x\le x^{2},\\ x^2 + \frac{1}{\sqrt{5}}x - 2, & x^{2}\le x\le y^1,\ \ \\ \frac{1}{2}x^2 - \frac{21}{10}, & y^1\le x.\ \ \end{array}\right. } \end{aligned}$$

One may readily verify that $f_1$ and $f_2$ are continuously differentiable. Both derivatives are monotonically increasing (piecewise linear) functions, and therefore $f_1$ and $f_2$ are convex. By the definition of curvature, it is also clear that both functions have minimum curvature *μ =1*, and maximum curvature *L=2*, as required. Note also that $f(x^1) = f(0) = f_1(0) - f_2(0) = 1$, and that the minimum is $f(x_\star ) = 0$.

We now perform one iteration (*N=1*) of Algorithm 2 on $f = f_1 - f_2$, starting from $x^1 =0$. Since $g_2^1 = \nabla f_2(x^1) = 0$, step 2 of the algorithm reduces to

$$\begin{aligned} y^1 = \arg \min f_1(x) = -\frac{1}{\sqrt{5}}, \end{aligned}$$

as required. Thus the boosted step with *α = 1* yields $x^2 = -\frac{2}{\sqrt{5}}$, as required.

It remains to verify that the bound in Theorem 1 holds with equality for this example. To this end, note that

$$\begin{aligned} \min _{1\le k\le N+1}\frac{\left\| \nabla f(x^k)\right\| ^2}{L} = \frac{1}{2}\min \left\{ \frac{4}{5}, \frac{4}{5}\right\} = \frac{2}{5} = \frac{(f(x^1)-f^\star ) }{(1+\kappa \alpha )N + \frac{1}{2(1-\kappa )}}, \end{aligned}$$

as required.

### Outline of this paper

Theorem 1 is proved below in Sect. 3 using the semidefinite programming (SDP) performance estimation approach, as pioneered by Drori and Teboulle [8]. More precisely, we use the refined version from [20, 21] to allow for tight analysis. Thus, we will first review the performance estimation method in some detail in Sect. 2. In Sect. 4 we show how one may use the analysis from Sect. 3 to analyze a variant of boosted DCA with backtracking, as opposed to a fixed step length *α > 0*. The advantage of the backtracking scheme is that one does not need to know the curvature parameters to select the step length. One of the attractive features of the DCA is that it contains some well-known algorithms as special cases, like gradient descent, and proximal gradient descent; see e.g. [16]. In Sect. 5, we investigate the implications of our analysis for the boosted DCA in the special case of gradient descent. In particular, if the objective satisfies a quadratic Łojasiewicz condition (also often referred to as Polyak-Łojasiewicz), we present results on the rate of linear convergence. Section 6 concludes with open questions and a further discussion of related literature.

## Performance estimation

A key step to the desired convergence results consists in formulating an abstract optimization problem that aims to find the worst-case input $f_1 \in {\mathcal {F}}_{\mu _1,L_1}({\mathbb {R}}^n)$ and $f_2 \in {\mathcal {F}}_{\mu _2,L_2}({\mathbb {R}}^n)$ for Algorithm 2, where we view $\mu _1 \ge 0,\mu _2\ge 0,L_1 \in [\mu _1,\infty ],L_2 \in [\mu _2,\infty ]$ as given parameters:

2 $$\begin{aligned} \begin{aligned} \max&\ \left( \min _{1\le k\le N+1} \left\| g_1^k-g_2^k\right\| ^2\right) \\&\ f_1\in {\mathcal {F}}_{\mu _1,L_1}({\mathbb {R}}^n), f_2\in {\mathcal {F}}_{\mu _2,L_2}({\mathbb {R}}^n)\\&f_1(x)-f_2(x)\ge f^\star \ \ \ \forall x\in {\mathbb {R}}^n\\&f_1(x^1)-f_2(x^1)-f^\star \le \Delta \\&\ g_1^{N+1}, g_2^{N+1}, x^{N+1}, \ldots , x^2 \ \text {are generated by Algorithm~2 w.r.t.}\ f_1, f_2, x^1 \\&\ x^1\in {\mathbb {R}}^n, \end{aligned} \end{aligned}$$

where $f_1, f_2$ and $x^k, g_1^k, g_2^k$ ($k\in \{1,..., N+1\}$) are decision variables, and $\Delta ,\mu _1,L_1,\mu _2,L_2, N$ are fixed parameters.

At first glance, this problem seems intractable, since we need to optimize over the infinite-dimensional function spaces ${\mathcal {F}}_{\mu _1,L_1}({\mathbb {R}}^n)$ and ${\mathcal {F}}_{\mu _2,L_2}({\mathbb {R}}^n)$. However, since we only reference $f_1$ and $f_2$ (and their subgradients) at the *N* iterates, we may reformulate these membership conditions as tractable interpolation conditions as in [20].

To this end, consider a finite index set *I* (that will correspond to iterates in our analysis), and given a set of triplets $\left\{ (x^k,g^k,f^k) \right\} _{k\in I}$ where $x^k \in {\mathbb {R}}^n$, $g^k\in {\mathbb {R}}^n$ and $f^k\in {\mathbb {R}}$. We are concerned with the following interpolation/extension result: for given $\mu \ge 0$ and $L \ge \mu$, does there exist a $f\in {\mathcal {F}}_{\mu , L}({\mathbb {R}}^n)$ such that $f(x^k)=f^k$, and $g^k\in \partial f(x^k)$ for all *k∈ I*. If yes, we say that $\left\{ (x^k,g^k,f^k) \right\} _{k\in I}$ is ${\mathcal {F}}_{\mu , L}({\mathbb {R}}^n)$-interpolable.

### Theorem 2

( [20], Theorem 4) Assume $\mu \ge 0$ and $L \in [\mu ,\infty ]$. The following statements are equivalent:

1. $$\left\{ (x^i,g^i,f^i) \right\} _{i\in I}$$ is $${\mathcal {F}}_{\mu , L}({\mathbb {R}}^n)$$-interpolable;
2. $$\forall i,j\in I$$:
  3 $$\begin{aligned} & \frac{1}{2(1-\frac{\mu }{L})}\left( \frac{1}{L}\left\| g^i-g^j\right\| ^2+\mu \left\| x^i-x^j\right\| ^2-\frac{2\mu }{L}\left\langle g^j-g^i,x^j-x^i\right\rangle \right) \nonumber \\ & \quad \le f^i-f^j-\left\langle g^j, x^i-x^j\right\rangle . \end{aligned}$$

Using this theorem, as well as the following lemma, we may reformulate (2) as an SDP.

### Lemma 2

([1], Lemma 2.1 (*Descent lemma*)) Let $f_1\in {\mathcal {F}}_{\mu _1, L_1}({{\mathbb {R}}^n})$ and $f_2\in {\mathcal {F}}_{\mu _2, L_2}({{\mathbb {R}}^n})$ and let $f=f_1-f_2$. If $g_1\in \partial f_1(x)$ and $g_2\in \partial f_2(x)$, then

$$\begin{aligned} f^\star \le f(x)-\tfrac{1}{2\left( L_1-\mu _2\right) }\Vert g_1-g_2\Vert ^2. \end{aligned}$$

To reformulate the performance estimation problem, we need some more notation to accommodate the two different types of points $x^k$ and $y^k$ generated by Algorithm 2. In particular, we introduce the vector variable $g_1(x^k)$ to correspond to a subgradient of $f_1$ at $x^k$, $g_2(y^k)$ that corresponds to a subgradient of $f_2$ at $y^k$, etc. Similarly, we introduce scalar variables $f_1(x^k)$ to correspond to the function value of the function $f_1$ at $x^k$, etc. Finally, for easy reference, we define a set of variables:

$$\begin{aligned} S:= \left\{ x^1,\ldots ,x^{N+1},y^1,\ldots ,y^{N}\right\} . \end{aligned}$$

We thus arrive at the following performance estimation problem:

4 $$\begin{aligned} \begin{aligned} \max&\ \left( \min _{1\le k\le N+1} \left\| g_1(x^k)-g_2(x^k)\right\| ^2\right) \\ \text{ s.t. } \ &\tfrac{1}{2(1-\tfrac{\mu _\ell }{L_\ell })}\left( \tfrac{1}{L_\ell }\left\| g_{\ell }(u)-g_{\ell }(v)\right\| ^2+\mu _\ell \left\| u-v\right\| ^2 -\tfrac{2\mu _\ell }{L_\ell }\left\langle g_{\ell }(v)-g_{\ell }(u),v-u\right\rangle \right) \le \\&\ \ \ \ \ f_{\ell }(u)-f_{\ell }(v)-\left\langle g_{\ell }(v), u-v\right\rangle \ \ \forall u,v \in S, \; \ell \in \{1,2\}\\&g_{1}(y^k)=g_{2}(x^k) \ \ k\in \left\{ 1, \ldots , N\right\} \\&x^{k+1}= y^k + \alpha (y^{k}-x^{k}) \ \ k\in \left\{ 1, \ldots , N\right\} \\&f_1(u)-f_2(u)-\frac{1}{2(L_1-\mu _2)}\Vert g_1(u)-g_2(u)\Vert ^2\ge f^\star \ \ \forall u \in S\\&f_1(x^1)-f_2(x^1)-f^\star \le \Delta , \end{aligned} \end{aligned}$$

where $S, \; g_{\ell }(u), f_\ell (u) \; (\ell \in {1,2}, u \in S)$ are decision variables, and where $\Delta ,\mu _1,L_1,\mu _2,L_2, N$ are fixed parameters.

By the *descent lemma* (Lemma 2), the constraints $f(x)\ge f^\star$ for each $x\in {\mathbb {R}}^n$ is replaced by

5 $$\begin{aligned} f_1(u)-f_2(u)-\frac{1}{2(L_1-\mu _2)}\Vert g_1(u)-g_2(u)\Vert ^2\ge f^\star \ \ \forall \; u \in S. \end{aligned}$$

The optimality conditions for the convex subproblem,

$$\begin{aligned} y^{k}\in \text {argmin}_{x\in {\mathbb {R}}^n} f_1(x)-\left( f_2(x^k)+\langle g_2^k, x-x^k\rangle \right) , \end{aligned}$$

are equivalent to $$ g_{1}(y^k)=g_{2}(x^k)$$ for some $$g_1(y^k)\in \partial {f_1}(y^{k})$$; see Theorem 3.63 in [7] (Fermat’s theorem). (For a more detailed discussion of criticality in D.C. programming, see e.g. [1, Section 2].)

The final problem (4) may be written as an SDP problem, since it is linear in the gram matrix of the vector variables, after using the equality constraints to reduce variables.

**Fig. 1:**
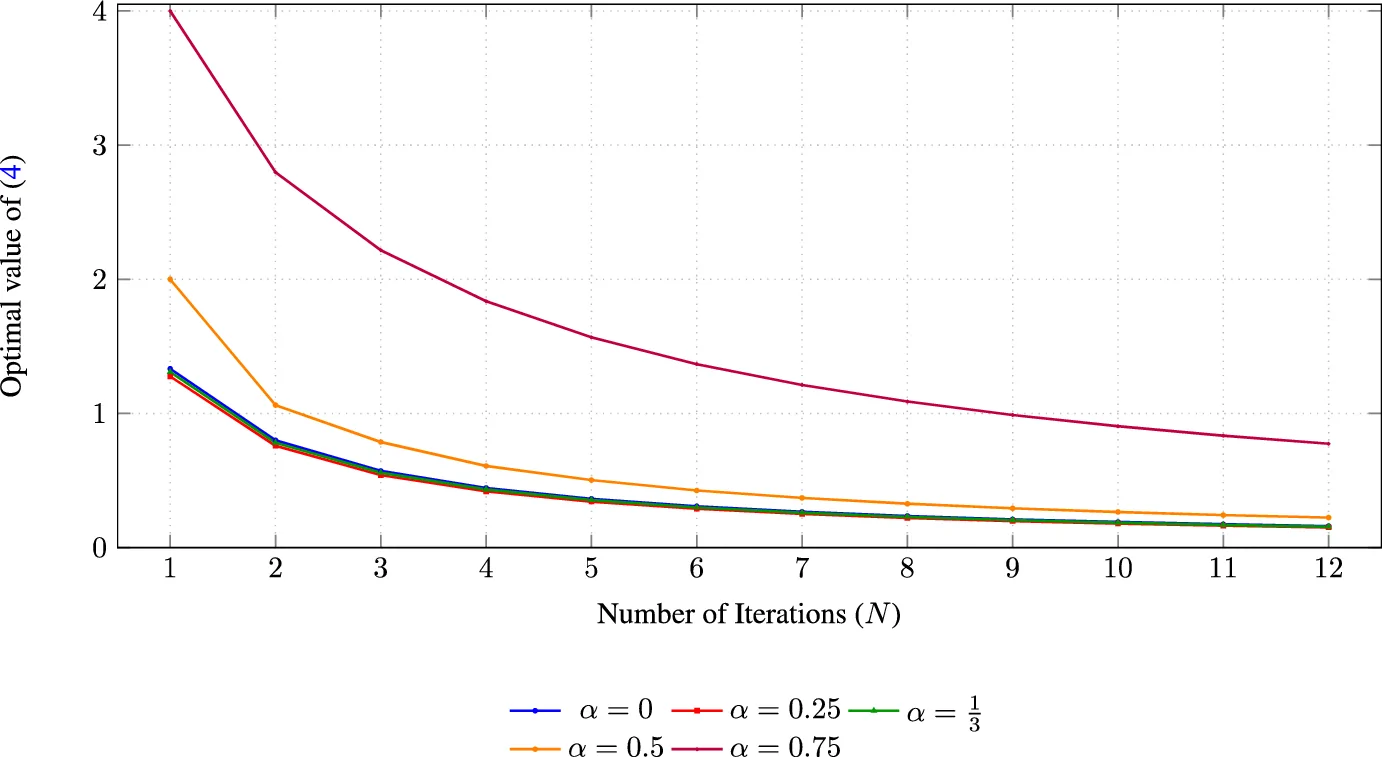
Performance bounds for DCA (*α =0*) and boosted DCA when $L_1=L_2=2$, and $\mu _1=1,\ \mu _2=0$

To illustrate this fact, we provide some graphs where the SDPs were solved numerically through MOSEK [15]. In Fig. 1 we consider the worst-case scenario after *N=12* iterations of Algorithm 2, for growing values of *α *, and for $L_1=L_2=2$, $\mu _1=1$, and $\mu _2=0$. Note that this choice of curvature parameters is not covered by the result in Theorem 1, since there it is assumed that $\mu _1 = \mu _2 > 0$. In the figure we see that some choices of *α * result in poorer rates of convergence than *α = 0* (the usual DCA).

**Fig. 2:**
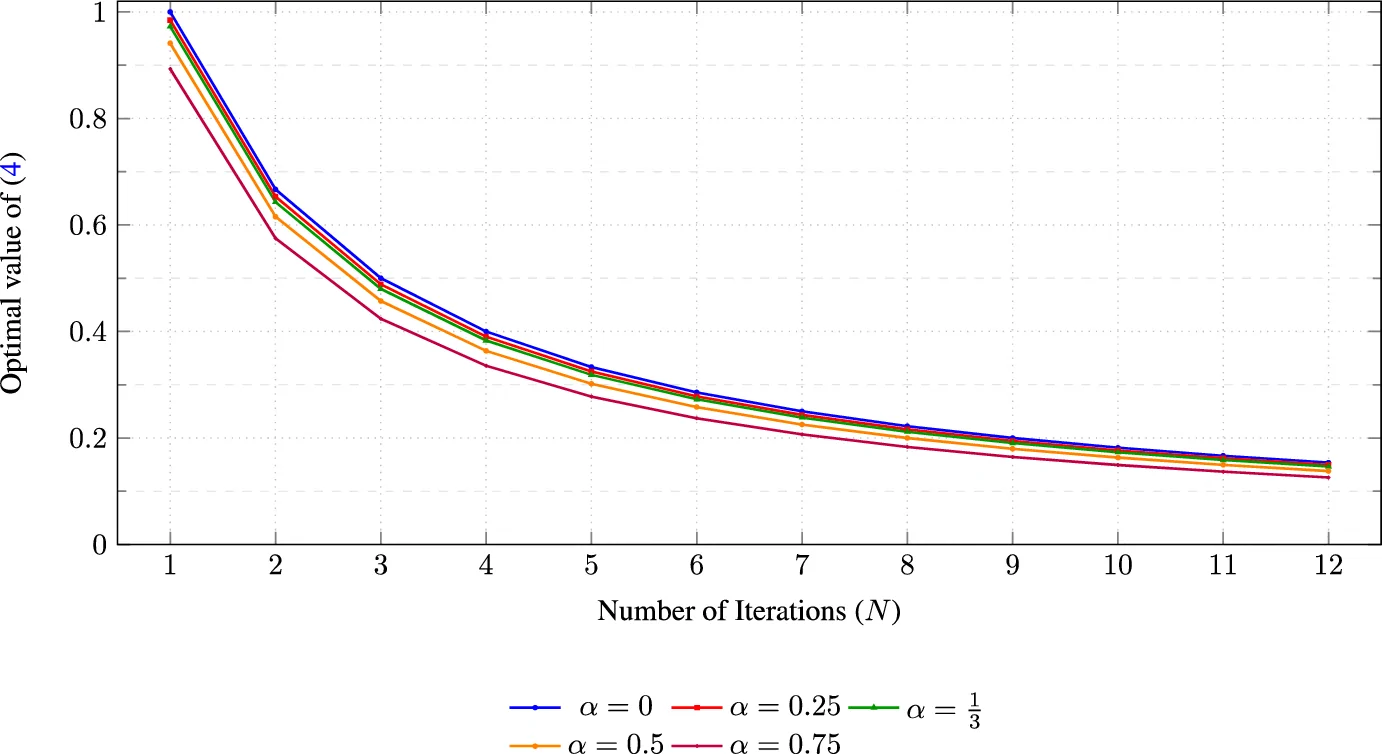
Performance bounds for DCA (*α =0*) and boosted DCA when $L_1=L_2=2$, and $\mu _1=0,\ \mu _2=1$

In Fig. 2, we again consider *N=12* iterations, but this time with the curvature parameters $L_1=L_2=2$, and $\mu _1=0,\ \mu _2=1$. Here, one does see improving rates of convergence for growing values of *α *, i.e. boosted DCA does outperform DCA here, although this is not predicted by Theorem 1, since $\mu _1=0$.

**Fig. 3:**
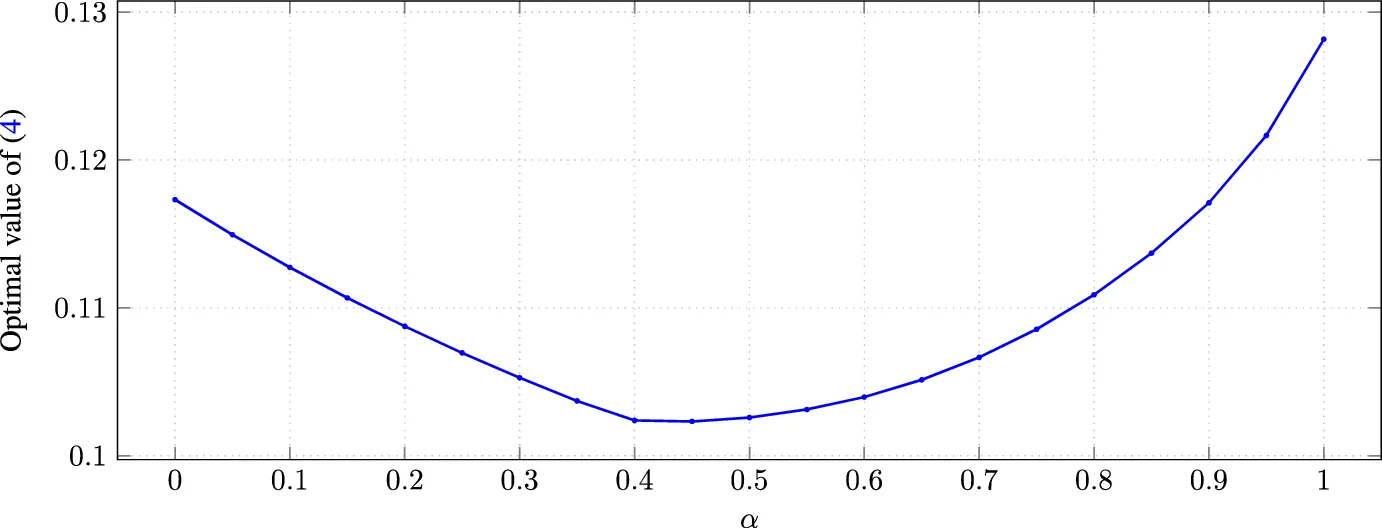
Performance bounds for DCA (*α =0*) and boosted DCA for the case that $L_1=1\ L_2=2$, and $\mu _1=0.5,\ \mu _2=0.25$ and *N=10*

In Fig. 3, we plot how the convergence criterion depends on *α *, for *N=10* iterations and parameter values $L_1=1,\ L_2=2$, and $\mu _1=0.5,\ \mu _2=0.25$. Note that the graph is a convex function of *α * with minimizer $\alpha ^\star \approx 0.4$. Also note that - when the value of *α * gets too large - the worst-case performance of DCA is actually better. Once again, the results in Fig. 3 are not covered by Theorem 1, since $\mu _1\ne \mu _2$ here.

### Adding the Polyak-Łojasiewicz (PŁ) inequality

We may also consider the DC problem (1) in the case where the objective satisfies the Polyak-Łojasiewicz (PŁ) inequality, defined as follows.

#### Definition 1

A differentiable function *f* is said to satisfy the PŁ inequality on $X\subseteq {\mathbb {R}}^n$ if there exists *η >0* such that

$$\begin{aligned} f(x)-f^\star \le \tfrac{1}{2\eta } \Vert \nabla f(x)\Vert ^2, \ \ \ \forall x\in X. \end{aligned}$$

Under the PŁ inequality assumption, every stationary point of *f* is a global minimizer, but it is a weaker assumption than convexity.

As done in the performance analysis of DCA in [1], one may incorporate the PŁ inequality in the SDP problem (4), by adding the constraints:

6 $$\begin{aligned} f_1(u) - f_2(u) -f^\star \le \tfrac{1}{2\eta } \Vert g_1(u) - g_2(u)\Vert ^2 \; \forall u \in S. \end{aligned}$$

Here we implicitly assume that the variables in *S* only take values in *X*.

Note that one could, instead of using (6), build on [17, Proposition 3.4] to obtain a more accurate picture for this class of functions (at the cost of much more complicated formulations). This is done in the numerical experiments below.

## Sublinear convergence of boosted DCA

In this section we give a proof of Theorem 1. The proof proceeds in the usual manner for SDP performance estimation: we provide suitable multipliers for the constraints of problem (4), and then aggregate the constraints with these multipliers to obtain the required inequality. The values of the multipliers in Table 1 below were obtained in a computer-assisted manner: after solving the (dual) SDP for different choices of the parameters, we could guess the analytic expressions for the multipliers, and subsequently verify the guesses in a rigorous manner. In other words, our proof of Theorem 1 was ‘discovered’ in a computer-assisted manner, but the proof itself is self-contained, and does not rely on any computational results.

### of Theorem 1

We decompose the proof into three parts:

1. Reformulate the desired statement.
2. Show that the reformulation can be proved using a simple one-iteration analysis and using induction.
3. Perform the one-iteration analysis.

**Part 1 of the proof: reformulation.** The main result follows if we prove the following inequality:

7 $$\begin{aligned} \begin{aligned}&\left[ \frac{1}{2}+\alpha \frac{\mu }{L}\right] \Vert g_1(x^1)-g_2(x^1)\Vert ^2 + \sum _{i=2}^N\left[ 1+\alpha \frac{\mu }{L}\right] \Vert g_1(x^i)-g_2(x^i)\Vert ^2\\&\quad +\left[ \frac{1-\frac{1}{2}\frac{\mu }{L}}{1-\frac{\mu }{L}}\right] \Vert g_1(x^{N+1})-g_2(x^{N+1})\Vert ^2\\ \le&L \left[ f_1(x^1)-f_2(x^1)-f_1(x^{N+1})+f_2(x^{N+1})+\frac{1}{2(L-\mu )}\Vert g_1(x^{N+1})-g_2(x^{N+1})\Vert ^2 \right] . \end{aligned} \end{aligned}$$

Indeed, using the upper bound on $f^\star$ from Lemma 2 with $x = x^{N+1}$, we get

$$\begin{aligned} \begin{aligned}&\left[ \frac{1}{2}+\alpha \frac{\mu }{L}\right] \Vert g_1(x^1)-g_2(x^1)\Vert ^2 + \sum _{i=2}^N\left[ 1+\alpha \frac{\mu }{L}\right] \Vert g_1(x^i)-g_2(x^i)\Vert ^2 \\&\quad + \left[ \frac{1-\frac{1}{2}\frac{\mu }{L}}{1-\frac{\mu }{L}}\right] \Vert g_1(x^{N+1})-g_2(x^{N+1})\Vert ^2 \\ \le&L \left[ f_1(x^1)-f_2(x^1)-f^\star \right] . \end{aligned} \end{aligned}$$

Then, lower-bounding the left-hand side by the smallest gradient norm observed, we obtain:

$$\begin{aligned} \begin{aligned} \min _{1\le i\le N+1}\left( \Vert g_1(x^i)-g_2(x^i)\Vert ^2\right) \left[ \frac{1}{2}+\alpha \frac{\mu }{L} +\sum _{i=2}^N\left( 1+\alpha \frac{\mu }{L}\right) +\frac{1-\frac{1}{2}\frac{\mu }{L}}{1-\frac{\mu }{L}}\right] \\&\quad \le L \left[ f_1(x^1)-f_2(x^1)-f^\star \right] , \end{aligned} \end{aligned}$$

where $\left[ \frac{1}{2}+\alpha \frac{\mu }{L}+\sum _{i=2}^N\left( 1+\alpha \frac{\mu }{L}\right) +\frac{1-\frac{1}{2}\frac{\mu }{L}}{1-\frac{\mu }{L}}\right] =(1+\kappa \alpha )N + \frac{1}{2(1-\kappa )}$, yielding the desired result.

**Part 2 of the proof: reducing to single iteration analysis.** We may prove that the required result (7) holds by proving that the following inequality holds for a given iteration $k \in {\mathbb {N}}$:

8 $$\begin{aligned} f(x^{k+1})+\frac{1}{2L}\Vert g_1(x^{k+1})-g_2(x^{k+1})\Vert ^2+ \frac{1}{L} \left[ \frac{1}{2} +\alpha \frac{\mu }{L}\right] \Vert g_1(x^k)-g_2(x^k)\Vert ^2 \le f(x^k). \end{aligned}$$

We will verify in part 3 of this proof that (12) indeed holds at each iteration, but first show that it implies the required result (7). Assuming now that (12) holds for any $k \in {\mathbb {N}}$, we will prove by induction that, for any *k ≥ 2*, one has

9 $$\begin{aligned} \begin{aligned}&f(x^k)+\frac{1}{L}\left[ \frac{1}{2}+\alpha \frac{\mu }{L}\right] \Vert g_1(x^1)-g_2(x^1)\Vert ^2+ \sum _{i=2}^{k-1}\frac{1}{L}\left[ 1+\alpha \frac{\mu }{L}\right] \Vert g_1(x^i)-g_2(x^i)\Vert ^2 \\&\quad +\frac{1}{2L}\Vert g_1(x^k)-g_2(x^k)\Vert ^2 \\ \le&f(x^1), \end{aligned} \end{aligned}$$

with the usual convention that the empty sum is zero (for *k=2*).

The base case for the induction argument is obtained by setting *k=1* in (12). For the induction step, let *k≥ 2* and assume that (9) holds. Then applying inequality (12) to lower bound $f(x^k)$ yields

$$\begin{aligned} \begin{aligned}&f(x^{k+1})+\frac{1}{L}\left[ \frac{1}{2}+\alpha \frac{\mu }{L}\right] \Vert g_1(x^1)-g_2(x^1)\Vert ^2 +\sum _{i=2}^{k}\frac{1}{L}\left[ 1+\alpha \frac{\mu }{L}\right] \Vert g_1(x^i)-g_2(x^i)\Vert ^2\\&\quad +\frac{1}{2L}\Vert g_1(x^{k+1})-g_2(x^{k+1})\Vert ^2 \\ \le&f(x^1). \end{aligned} \end{aligned}$$

Thus we have shown that (9) holds for all *k ≥ 2*. For *k=N*, we therefore obtain

10 $$\begin{aligned} \begin{aligned}&\frac{1}{L}\left[ \frac{1}{2}+\alpha \frac{\mu }{L}\right] \Vert g_1(x^1)-g_2(x^1)\Vert ^2 +\sum _{i=2}^{N}\frac{1}{L}\left[ 1+\alpha \frac{\mu }{L}\right] \Vert g_1(x^i)-g_2(x^i)\Vert ^2\\&\quad +\frac{1}{2L}\Vert g_1(x^{N+1})-g_2(x^{N+1})\Vert ^2 \\ \le&f(x^1)-f(x^{N+1}). \end{aligned} \end{aligned}$$

Proving (7) requires lower-bounding

$$\begin{aligned} f(x^1)-f(x^{N+1})+\frac{1}{2(L-\mu )}\Vert g_1(x^{N+1})-g_2(x^{N+1})\Vert ^2,\end{aligned}$$

as opposed to $f(x^1)-f(x^{N+1})$. To adjust the bound (15) to get to the desired inequality, we simply add $\frac{1}{2(L-\mu )}\Vert g_1(x^{N+1})-g_2(x^{N+1})\Vert ^2$ on both sides; this leads directly to (7) (after scaling by *L*).

**Part 3 of the proof: performing the single iteration analysis.** We now verify that (12) indeed holds at each iteration *k*.

Recall that $f_1,f_2 \in {\mathcal {F}}_{\mu ,L}({\mathbb {R}}^n)$, and therefore, by (3), satisfy

11 $$\begin{aligned} Q_\ell (u,v) \le 0, \quad \text{( }u,v \in {\mathbb {R}}^n\text{, } \ell \in \{1,2\}\text{) }, \end{aligned}$$

where we define

$$\begin{aligned} Q_\ell (u,v)= & \tfrac{1}{2(1-\tfrac{\mu }{L})}\left( \tfrac{1}{L}\left\| g_{\ell }(u)-g_{\ell }(v)\right\| ^2+\mu \left\| u-v\right\| ^2 -\tfrac{2\mu }{L}\left\langle g_{\ell }(v)-g_{\ell }(u),v-u\right\rangle \right) \\ & - f_{\ell }(u)+f_{\ell }(v)+\left\langle g_{\ell }(v), u-v\right\rangle , \\ \end{aligned}$$

and where $g_\ell (u)$ denotes a subgradient of $f_\ell$ at *u*, as before.

We now consider the inequalities (11) for specific choices of (*u*, *v*) as listed in Table 1, together with corresponding multipliers, $\lambda _{\ell ,u,v}$, also listed in the table.

**Table 1:** Multipliers for the interpolation constraints $Q_\ell (u,v) \le 0$ for a fixed index *k* and specific choices for (*u*, *v*)

(*u*, *v*)	$(x^k,y^k)$	$(y^k,x^k)$	$(x^{k+1},y^k)$	$(y^k,x^{k+1})$	$(x^{k+1},x^k)$
*ℓ *	1	1	1	1	2
$\lambda _{\ell ,u,v}$	$1 + \frac{2\alpha \mu }{L}$	$\frac{2\alpha \mu }{L}$	$\frac{1}{\alpha } - 1$	$\frac{1}{\alpha }$	1

Note that all the multipliers in the table are nonnegative if $\alpha \in (0,1]$. Multiplying each inequality from (11) by the corresponding nonnegative multiplier from Table 1 for the specific choice of (*u*, *v*) listed in the table, and summing, we obtain the inequality:

$$\begin{aligned} \begin{aligned} 0\ge&f_1(x^{k+1})-f_2(x^{k+1})-f_1(x^k)+f_2(x^k)+\tfrac{1}{2L} \Vert g_1(x^{k+1})-g_2(x^{k+1})\Vert ^2\\&\quad +\tfrac{1}{L} \left( \frac{1}{2}+\alpha \frac{\mu }{L}\right) \Vert g_1(x^k)-g_2(x^k)\Vert ^2\\&+\tfrac{1}{2 \alpha L (L-\mu ) (\alpha \mu +2 (1-\alpha ) L)} \\&\quad \times \Vert (\alpha \mu -2 (\alpha -1) L)g_1(x^{k+1})+L(\alpha -2)g_2(x^k)+\alpha (L-\mu )g_2(x^{k+1})\\&\quad +\alpha L (-\alpha \mu +\mu +L)(x^k-y^k)\Vert ^2\\&+\tfrac{\alpha L-2\mu }{2(L-\mu )(2L(\alpha -1)-\alpha \mu )}\Vert -g_2(x^k)+g_2(x^{k+1})+(L+\alpha \mu )(x^k-y^k)\Vert ^2\\&+\tfrac{\mu (L+2\alpha L+2\alpha \mu )}{2L^2(L-\mu )}\Vert g_1(x^k)-g_2(x^k)-L(x^k-y^k)\Vert ^2. \end{aligned} \end{aligned}$$

The coefficients of the last three terms are nonnegative when $0<\alpha \le \min \left\{ 2\frac{\mu }{L}, \frac{1}{1-\frac{1}{2}\frac{\mu }{L}}\right\} =2\kappa$. Removing the last three nonnegative terms on the right-hand-side, one arrives to the desired

$$\begin{aligned} \begin{aligned} 0 \ge&f_1(x^{k+1})-f_2(x^{k+1})-f_1(x^k)+f_2(x^k)+\tfrac{1}{2L} \Vert g_1(x^{k+1})-g_2(x^{k+1})\Vert ^2\\&\quad +\tfrac{1}{L} \left( \frac{1}{2}+\alpha \frac{\mu }{L}\right) \Vert g_1(x^k)-g_2(x^k)\Vert ^2\\ =&f(x^{k+1})-f(x^k)+\tfrac{1}{2L} \Vert g_1(x^{k+1})-g_2(x^{k+1})\Vert ^2\\&\quad +\tfrac{1}{L} \left( \frac{1}{2}+\alpha \frac{\mu }{L}\right) \Vert g_1(x^k)-g_2(x^k)\Vert ^2, \end{aligned} \end{aligned}$$

and the proof therefore holds when $0<\alpha \le \min \left\{ 2\kappa ,1\right\}$. *□ *

## Sublinear convergence of boosted DCA with backtracking

By Theorem 1, the optimal choice for fixed *α > 0* in Algorithm 2 is $\alpha = \min \{1,2\kappa \}$. However, this requires knowledge of the parameter $\kappa = \mu /L$, while the usual DCA (with *α = 0*) does not require knowledge of the parameters *μ * and *L*.

By leveraging our proof technique for Theorem 1, we may also analyse a variant of boosted DCA where the parameters *μ * and *L* are not known, but only estimated at each step, and where we use backtracking to find a suitable step length.

Backtracking analysis dates back to the work by Armijo [6] and Goldstein [10] (see, e.g., [6, Corollary 2]), and our aim here is only to show that this type of (standard) analysis may be combined with our proof technique to obtain a parameter-free variant of Algorithm 2.

With reference to Algorithm 3, the main ideas of the backtracking analysis are as follows:

- At each iteration *k*, we have estimates $\mu ^{(k)}$ of *μ * and $L^{(k)}$ of *L* respectively.
- By the proof of Theorem 1, the following inequality holds at each iteration *k* if using the correct values of $(\mu ,L,\alpha )$:
  12 $$\begin{aligned} f(x^{k+1})+\frac{1}{2L}\Vert g_1(x^{k+1})-g_2(x^{k+1})\Vert ^2+ \frac{1}{L} \left[ \frac{1}{2} +\alpha \frac{\mu }{L}\right] \Vert g_1(x^k)-g_2(x^k)\Vert ^2 \le f(x^k). \end{aligned}$$
  At each iteration, we test if it also holds for the estimates $\mu ^{(k)}$, $L^{(k)}$ and $\alpha ^{(k)}:= \min \{1,2\mu ^{(k)}/L^{(k)}\}$. If not, we adjust these estimates as detailed in step 6 of Algorithm 3. In particular, we use a fixed parameter *β > 1* to decrease $\mu ^{(k)}$ via $\mu ^{(k)}\leftarrow \mu ^{(k)}/\beta$, and increase $L^{(k)}$ via $L^{(k)}\leftarrow \beta L^{(k)}$, if needed.
- In the statement of Theorem 3 below, the quantity ${\bar{K}}$ bounds the maximum number of parameter updates in step 6 of Algorithm 3. Thus, for each iteration *k*, one has $L^{(k)}\le {\bar{L}}:=\beta ^{{\bar{K}}}\max \{L^{(0)}, L\}$ and $\mu ^{(k)}\ge {\bar{\mu }}:= \beta ^{-{\bar{K}}}\min \{\mu ^{(0)},\mu \}$. Thus, we also have $\alpha ^{(k)}\ge {\bar{\alpha }}:=\min \left\{ 2\frac{{\bar{\mu }}}{{\bar{L}}},1\right\}$ and $\kappa \ge {\bar{\kappa }}:=\frac{{\bar{\mu }}}{{\bar{L}}}$.

**Algorithm 3:**
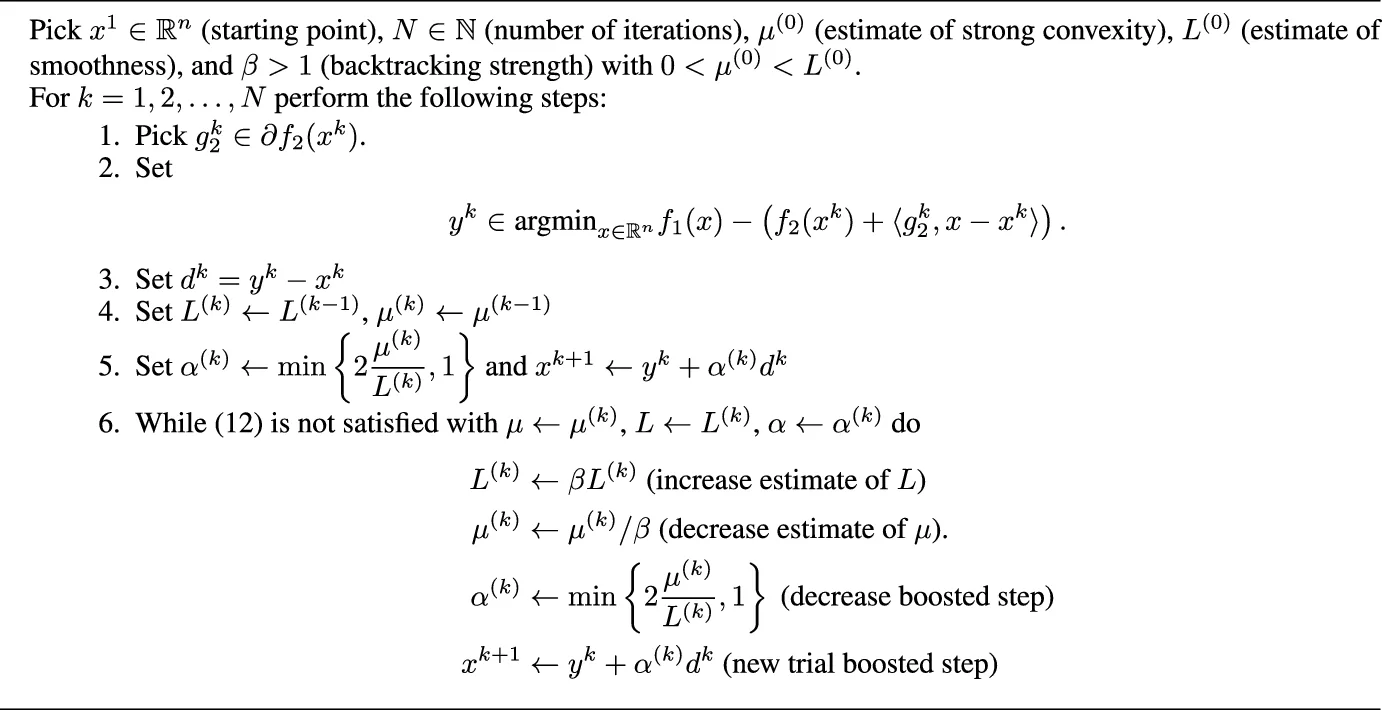
Boosted DCA with backtracking

The following theorem is a direct analog of Theorem 1, but for the worst-case analysis of the backtracking Algorithm 3.

### Theorem 3

Let $f_1\in {\mathcal {F}}_{\mu , L}({{\mathbb {R}}^n})$ and $f_2\in {\mathcal {F}}_{\mu , L}({{\mathbb {R}}^n})$, $0 \le \mu< L < \infty$. After *N* iterations of boosted DCA with backtracking (Algorithm 3) with initial guesses $0<\mu ^{(0)}<L^{(0)}$ and backtracking parameter *β >1*, one has,

$$\begin{aligned} \min _{1\le k\le N+1}\frac{\left\| g_1^k-g_2^k\right\| ^2}{{\bar{L}}}\le \frac{(f(x^1)-f^\star ) }{(1+{\bar{\kappa }}{\bar{\alpha }})N + \frac{1}{2(1-{\bar{\kappa }})}}, \end{aligned}$$

where $${\bar{K}}:= \max \left\{ 0,\lceil \log _{\beta }\left( \beta L/L^{(0)}\right) \rceil , \lceil \log _{\beta }\left( \beta \mu ^{(0)}/\mu \right) \rceil \right\} $$, $${\bar{L}}:=\beta ^{{\bar{K}}}\max \{L^{(0)}, L\}$$, $${\bar{\mu }}:= \beta ^{-{\bar{K}}}\min \{\mu ^{(0)},\mu \}$$, $${\bar{\alpha }}:=\min \left\{ 2\frac{{\bar{\mu }}}{{\bar{L}}},1\right\} $$, and $${\bar{\kappa }}:=\frac{{\bar{\mu }}}{{\bar{L}}}$$.

**Proof:** At each iteration *k*, it holds that

13 $$\begin{aligned} & f(x^{k+1})+\frac{1}{2L^{(k)}}\Vert g_1(x^{k+1})-g_2(x^{k+1})\Vert ^2+ \frac{1}{L^{(k)}} \left[ \frac{1}{2} +\alpha ^{(k)} \frac{\mu ^{(k)}}{L^{(k)}}\right] \Vert g_1(x^k)-g_2(x^k)\Vert ^2\nonumber \\ & \quad \le f(x^k). \end{aligned}$$

By definition of ${\bar{L}}\ge L^{(k)}$, ${\bar{\mu }}\le \mu ^{(k)}$, and ${\bar{\alpha }}\le \alpha ^{(k)}$, we get

14 $$\begin{aligned} & f(x^{k+1})+\frac{1}{2{\bar{L}}}\Vert g_1(x^{k+1})-g_2(x^{k+1})\Vert ^2+ \frac{1}{{\bar{L}}} \left[ \frac{1}{2} +{\bar{\alpha }} \frac{{\bar{\mu }}}{{\bar{L}}}\right] \Vert g_1(x^k)-g_2(x^k)\Vert ^2\nonumber \\ & \quad \le f(x^k), \end{aligned}$$

which is satisfied at all iterations (given that after at most ${\bar{K}}$ updates of the Lipschitz and strong convexity estimates, the corresponding values are both valid smoothness and strong convexity parameters for the problem at hand).

By induction, we thereby also get:

$$\begin{aligned} \begin{aligned}&f(x^{k+1})+\frac{1}{{\bar{L}}}\left[ \frac{1}{2}+{\bar{\alpha }}\frac{{\bar{\mu }}}{{\bar{L}}}\right] \Vert g_1(x^1)-g_2(x^1)\Vert ^2+\sum _{i=2}^{k} \frac{1}{{\bar{L}}}\left[ 1+{\bar{\alpha }}\frac{{\bar{\mu }}}{{\bar{L}}}\right] \\&\quad \Vert g_1(x^i)-g_2(x^i)\Vert ^2+\frac{1}{2{\bar{L}}}\Vert g_1(x^{k+1})-g_2(x^{k+1})\Vert ^2 \\ \le&f(x^1). \end{aligned} \end{aligned}$$

For *k=N*, we therefore obtain

15 $$\begin{aligned} \begin{aligned}&\frac{1}{{\bar{L}}}\left[ \frac{1}{2}+{\bar{\alpha }}\frac{{\bar{\mu }}}{{\bar{L}}} \right] \Vert g_1(x^1)-g_2(x^1)\Vert ^2+\sum _{i=2}^{N}\frac{1}{{\bar{L}}}\left[ 1+{\bar{\alpha }} \frac{{\bar{\mu }}}{{\bar{L}}}\right] \\&\quad \Vert g_1(x^i)-g_2(x^i)\Vert ^2+\frac{1}{2{\bar{L}}} \Vert g_1(x^{N+1})-g_2(x^{N+1})\Vert ^2 \\ \le&f(x^1)-f(x^{N+1}). \end{aligned} \end{aligned}$$

To arrive at the target inequality, we have to lower bound $f(x^1)-f^\star$. Since the objective function is also ${\bar{L}}$-smooth and ${\bar{\mu }}$-strongly convex, we can use the trick from the proof of Theorem 1, and instead lower bound

$$\begin{aligned} f(x^1)-f(x^{N+1})+\frac{1}{2({\bar{L}}-{\bar{\mu }})}\Vert g_1(x^{N+1})-g_2(x^{N+1})\Vert ^2.\end{aligned}$$

To adjust the bound (15) to get to the desired inequality, we simply add $\frac{1}{2({\bar{L}}-{\bar{\mu }})}\Vert g_1(x^{N+1})-g_2(x^{N+1})\Vert ^2$ on both sides. *□ *

## Consequences for gradient descent

In this section we look at the implications of our approach in the special case of gradient descent under the PŁ assumption. As will become clear, our approach allows for an improved analysis for *L*-smooth functions that meet the PŁ condition. Of course, one may also study performance analysis of gradient descent for such functions directly, as was done in [2], but we will show improved results via the boosted DCA analysis.

Consider the following optimization problem

$$\begin{aligned} \inf _{x\in {\mathbb {R}}^n} \ f(x) \end{aligned}$$

where *f* is lower-bounded by $f^\star$ and is an *L*-smooth function on ${\mathbb {R}}^n$ with *L<∞ *, that is $f\in {\mathcal {F}}_{-L,L}({\mathbb {R}}^n)$ and differentiable. It is easily seen that the function *f* can be written as

$$\begin{aligned} f(x):=\frac{L}{2}\Vert x\Vert ^2-\left( \frac{L}{2}\Vert x\Vert ^2-f(x)\right) = f_1(x) - f_2(x), \end{aligned}$$

where we define $f_1(x):=\frac{L}{2}\Vert x\Vert ^2$ and $f_2(x):=\frac{L}{2}\Vert x\Vert ^2-f(x)$. Both functions $f_1$ and $f_2$ are convex since *f* is *L*-smooth. If we solve the subproblem of the DCA (Algorithm 1) at iteration *k*, namely

$$\begin{aligned} x^{k+1}=\arg \min _x\ \frac{L}{2}\Vert x\Vert ^2-\left( \frac{L}{2}\left\| x^k\right\| ^2-f(x^k)\right) -\left\langle {L}x^k-\nabla f(x^k), x-x^k\right\rangle , \end{aligned}$$

we get

$$\begin{aligned} x^{k+1}=x^k-\frac{1}{L}\nabla f(x^k), \end{aligned}$$

which is exactly the gradient descent method with the classical step length $\frac{1}{L}$. Similarly, the boosted DCA (Algorithm 2) corresponds to gradient descent with constant step lengths $(1+\alpha )\frac{1}{L}$. This allows us to consider longer step lengths than 1/*L*.

We may now adapt the SDP performance analysis problem (4) for the special case where $$f_1(x) = \frac{L}{2}\Vert x\Vert ^2$$ and $$f_2 \in {\mathcal {F}}_{0, 2L}({{\mathbb {R}}^n})$$, i.e. $$\mu _2 = 0$$ and $$L_2 = 2L$$. Note that we now have $$ g_1(x) = Lx, $$ so that several constraints in (4) simplify. In particular, one may readily derive the following linear convergence result for boosted DCA under the PŁ inequality.

### Theorem 4

Let $f_1(x)=\frac{L}{2}\Vert x\Vert ^2$, $f_2\in {\mathcal {F}}_{0, 2L}({{\mathbb {R}}^n})$ and $f(x)=f_1(x)-f_2(x)$. If *f* satisfies PŁ inequality on $X=\{x: f(x)\le f(x^1)\}$ with modulus $0<\eta \le L$, then for $x^1, x^2$ from boosted DCA with given $\alpha \in \left[ 0, \frac{\sqrt{\kappa ^2 + 2\kappa +4}-\kappa }{2+ \kappa } \right]$, if $\kappa =\frac{\eta }{L}$ we have

$$\begin{aligned} \frac{f(x^2)-f^\star }{f(x^1)-f^\star }\le \beta < 1, \end{aligned}$$

where

16 $$\begin{aligned} \beta := \kappa \alpha ^2 - \frac{\kappa (2-2\kappa )}{\kappa +2}\alpha + \frac{2-2\kappa }{2+\kappa }. \end{aligned}$$

### Proof

We assume w.l.o.g. that $f^\star =0$ and $x^1=0$, so that $f_1(x^1) = 0$. This implies that $x^2 = y^1 + \alpha (y^1-x^1) = (1+\alpha )y^1$. Moreover, the optimality conditions for the DCA subproblem that yields $y^1$ imply

$$\begin{aligned} g_1(y^1) = -g_2(x^1) \Longleftrightarrow Ly^1 = - g_2(x^1). \end{aligned}$$

Thus $x^2 = - \frac{1+\alpha }{L}g_2(x^1)$, and therefore

17 $$\begin{aligned} f_1(x^2) = \frac{L}{2}\left\| \frac{1+\alpha }{L}g_2(x^1)\right\| ^2. \end{aligned}$$

Our proof relies on the following identity, that may be verified through direct calculation:

18 $$\begin{aligned}&{\left( \frac{L}{2}\left\| \frac{1+\alpha }{L}g_2(x^1)\right\| ^2 -f_2(x^2)-f^\star \right) }-\beta \left( {-f_2(x^1)-f^\star } \right) \end{aligned}$$

19 $$\begin{aligned}&+\alpha \left( f_2(x^1)-f_2(y^{1})+\left\langle g_2(y^{1}), \frac{1}{L}g_2(x^{1})\right\rangle -\tfrac{1}{4L}\left\| g_2(x^1)-g_2(y^{1})\right\| ^2\right) \end{aligned}$$

20 $$\begin{aligned}&+ \left( \frac{(2-\kappa )}{\kappa +2}\alpha + \frac{2}{\kappa +2}\right) \left( f_2(y^1)-f_2(x^{1})-\left\langle g_2(x^{1}), \frac{1}{L}g_2(x^{1})\right\rangle -\tfrac{1}{4L}\left\| g_2(x^1)-g_2(y^{1})\right\| ^2\right) \end{aligned}$$

21 $$\begin{aligned}&+\left( f_2(x^2)-f_2(y^{1})-\left\langle g_2(y^{1}), \frac{1+\alpha }{L}g_2(x^1)-\frac{1}{L}g_2(x^{1})\right\rangle -\tfrac{1}{4L}\left\| g_2(x^2)-g_2(y^{1})\right\| ^2\right) \end{aligned}$$

22 $$\begin{aligned}&+\left( -\kappa \alpha ^2 - \frac{2\kappa ^2}{\kappa +2}\alpha + \frac{2\kappa }{\kappa +2}\right) \left( \frac{1}{2\eta }\left\| g_2(x^1)\right\| ^2+f_2(x^1)\right) \end{aligned}$$

23 $$\begin{aligned}&+\left( \frac{\kappa (2\alpha +1)}{\kappa +2}\right) \left( \frac{1}{2\eta }\left\| g_2(x^1)-g_2(y^1)\right\| ^2-\frac{L}{2}\left\| \frac{1}{L}g_2(x^1)\right\| ^2+f_2(y^1)\right) \nonumber \\&=-\frac{1}{4L}\Vert g_2(x^2)-g_2(y^1)\Vert ^2. \end{aligned}$$

By (17), and since $f_1(x^1) = 0$, the first line (iazu) in the identity may be rewritten as:

$$\begin{aligned} f(x^2)-f^\star - \beta \left( f(x^1)-f^\star \right) = \frac{L}{2}\left\| \frac{1+\alpha }{L}g_2(x^1)\right\| ^2 -f_2(x^2)-f^\star -\beta \left( {-f_2(x^1)-f^\star } \right) . \end{aligned}$$

We will argue that all the terms (19), (20), (21), (22), and (23) are all nonnegative. The identity will then imply

$$\begin{aligned}f(x^2)-f^\star - \beta \left( f(x^1)-f^\star \right) \le -\frac{1}{4L}\Vert g_2(x^2)-g_2(y^1)\Vert ^2 \le 0,\end{aligned}$$

so that the outer inequality yields the required result.

To this end, note that (19) corresponds the interpolation constraint for $f_2$ at $(u,v) = (x^1,y^1)$; more precisely, (19) equals the quantity $- \alpha {\mathcal {Q}}_2(x^1,y^1)$ in (11) for $(u,v) = (x^1,y^1)$. Since the interpolation conditions require ${\mathcal {Q}}_2(x^1,y^1) \le 0$, one has $- \alpha {\mathcal {Q}}_2(x^1,y^1) \ge 0$, as required. Similarly, (20) corresponds the interpolation constraint for $f_2$ at $(u,v) = (y^1,x^1)$ with nonnegative multiplier $\left( \frac{(2-\kappa )}{\kappa +2}\alpha + \frac{2}{\kappa +2}\right)$, and (21) to the interpolation constraint for $f_2$ at $(u,v) = (x^2,y^1)$.

Line (22) corresponds to the PŁ inequality (6) at $x^1$, and the multiplier $\left( -\kappa \alpha ^2 - \frac{2\kappa ^2}{\kappa +2}\alpha + \frac{2\kappa }{\kappa +2}\right)$ is nonnegative precisely if *α * is upper bounded as in the statement of the theorem. Similarly, (23) corresponds to the PŁ inequality (6) at $y^1$ with a nonnegative multiplier. This completes the proof.

*□*

Note that, under the conditions of the theorem, one has $f(x^2) \le f(x^1)$. As a consequence, all iterates lie in the set $X=\{x: f(x)\le f(x^1)\}$ where the PŁ inequality holds. Thus BCDA converges at a linear rate with constant *β < 1* as defined in (16).

Note that the right hand side is a convex parabola in *α * with minimum at $\alpha ^* = \frac{1-\kappa }{2+\kappa }$. We immediately obtain the following corollary for gradient descent.

### Corollary 1

Let $f\in {\mathcal {F}}_{-L, L}({{\mathbb {R}}^n})$ satisfy the PŁ inequality on $\{x: f(x)\le f(x^1)\}$ with modulus *η *, then

$$\begin{aligned} x^2 = x^1 - \frac{1 + \alpha ^*}{L}\nabla f(x^1) \end{aligned}$$

satisfies

$$\begin{aligned} \frac{f(x^2)-f^\star }{f(x^1)-f^\star }\le \frac{4-\kappa ^3 - 3\kappa }{(2+\kappa )^2}, \end{aligned}$$

for $$\alpha ^* = \frac{1-\kappa }{2+\kappa }$$ and $$\kappa =\frac{\eta ,}{L}$$.

The optimal choice for the boosting parameter $\alpha ^* = \frac{1-\kappa }{2+\kappa }$ corresponds to the step length

$$\begin{aligned} \frac{1+\alpha ^*}{L} = \frac{3}{(2+\kappa )L}. \end{aligned}$$

This leads to the convergence bound

$$\begin{aligned} \frac{f(x^2)-f^*}{f(x^1)-f^*} \le \frac{4-\kappa ^3-3\kappa }{(2+\kappa )^2}. \end{aligned}$$

This step length improves on the previous best known step length, given by $\min \left\{ \frac{3}{2L}, \frac{2}{(1+\sqrt{\kappa })L}\right\}$, from [2, Corollary 1] for *L*-smooth convex functions. A direct comparison shows the new bound is tighter if and only if the condition number is in the range *0< κ < 1/4*.

**Fig. 4:**
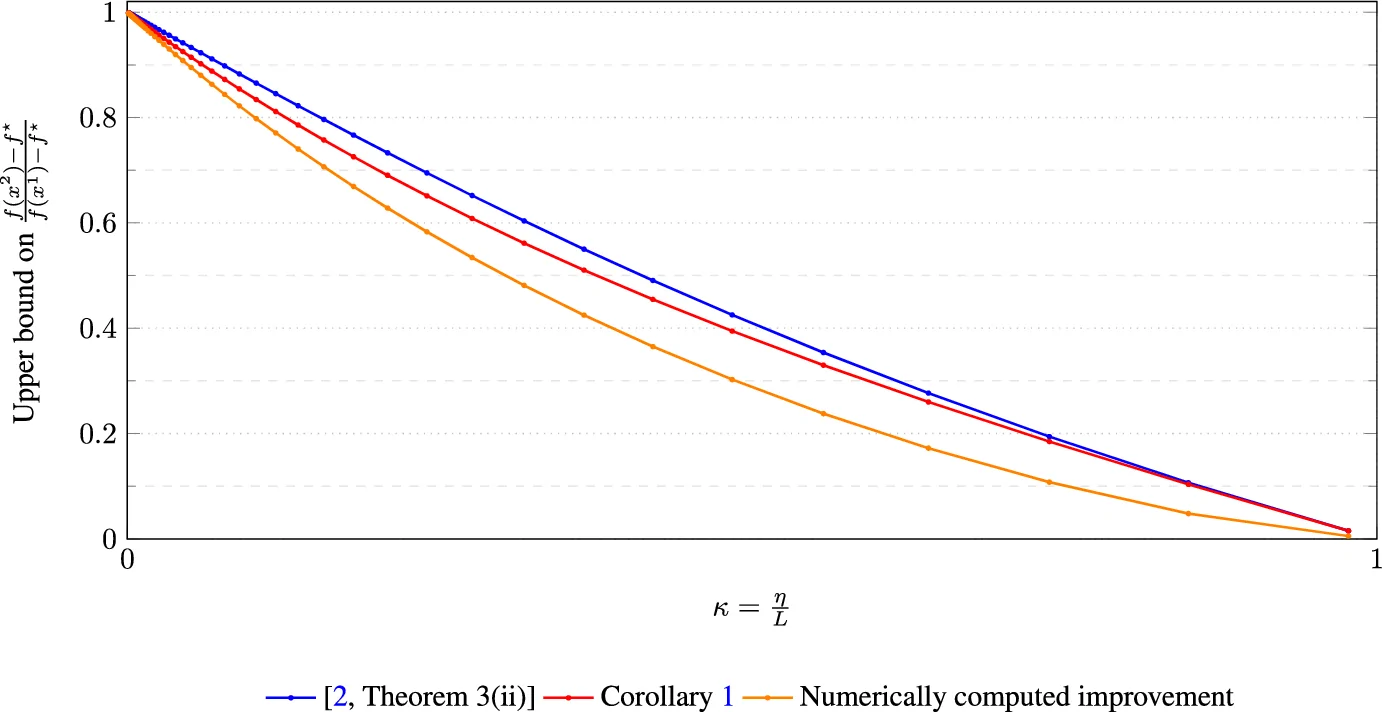
Comparisons between the bounds from Corollary 1 on $\frac{f(x^2)-f^\star }{f(x^1)-f^\star }$ for gradient descent with step-size $\frac{3}{(2+\kappa ) L}$ for a range of *κ *. The ‘numerically computed improvement’ uses the stronger characterization of smooth Łojasiewicz functions from [17, Proposition 3.4]

Figure 4 illustrates the bound from Corollary 1. The figure shows that the bound from Corollary 1 improves on the earlier bound from [2, Theorem 3(ii)] for the same step length. To show that the new bound is still not tight, we include another (numerically computed) bound in the figure that uses the stronger characterization of smooth Łojasiewicz functions from [17, Proposition 3.4]. We were unable, though, to obtain an analytical expression for the latter bound, and therefore omit further details here, especially since we believe the numerically computed bound is still not tight.

## Conclusion and discussion

In this paper we extended the SDP performance analysis for DCA by Abbaszadeh et al. [1] to the boosted DCA. Having said that, our main result in Theorem 1 only covers the case where both $f_1,f_2 \in {\mathcal {F}}_{\mu ,L}({\mathbb {R}}^n)$ for given *0< μ < L*. It would be interesting to obtain more detailed results with $f_1 \in {\mathcal {F}}_{\mu _1,L_1}({\mathbb {R}}^n)$ and $f_2 \in {\mathcal {F}}_{\mu _2,L_2}({\mathbb {R}}^n)$ where $\mu _1 \ne \mu _2$, etc, and where (one of) $L_1$ and $L_2$ may be infinite. (Such results were presented in [1].)

In related work, Rotaru et al. [16] extended the analysis in [1] to generalised DCA, where one allows $\mu _2 < 0$, as long as $\mu _1+\mu _2 \ge 0$. (It is known that this generalised DCA is still a descent method.) In the same work, Rotaru et al. [16] also consider the implications for the special case of the proximal gradient method, and obtain some improved worst-case convergence bounds. This is similar in spirit to the analysis in our paper, where we derived new results for the special case of gradient descent (Theorem 4). A natural question is to extend the analysis by Rotaru et al. [16] to the boosted case.

This work raises a few open questions:

1. In Theorem 1 and Theorem 4, the performance measure is a convex function of the boosted step length *α *. We also observe this numerically in Fig. 3, even though this is not covered by theory. A natural question is whether this is always the case.
2. Condition (6) was used to model the PŁ inequality. This condition is known to be necessary, but not sufficient, to characterize the desired function class in the performance analysis framework. While Sect. 5 shows that adding appropriate points to the discrete description of the function at hand allows improving upon known convergence results, it remains clear that (6) can also be refined. A natural (but complicated) candidate is provided in [17, Proposition 3.4] and is shown numerically to allow improving even more the results from Theorem 4 and Corollary 1 (using the refined discrete description of the function); see Fig. 4. The results from Theorem 4 and Corollary 1 are therefore not tight in general, and it remains unclear how to obtain analytical expressions of tight bounds.
3. As for now, it remains open to generalise the analysis to possibly include constraints, e.g., by allowing one of the Lipschitz constants to take the value *+∞ *. This is of practical interest, though, since the boosted DCA has been extended to allow linear constraints; see [5, 22]. Any progress in this direction would be welcome.
4. It would be desirable to derive a generalization of Theorem 1 where $f_1\in {\mathcal {F}}_{\mu _1, L_1}({{\mathbb {R}}^n})$ and $f_2\in {\mathcal {F}}_{\mu _2, L_2}({{\mathbb {R}}^n})$, and where one allows $\mu _1 \ne \mu _2$ and $L_1 \ne L_2$. However, we could only establish Theorem 1 for the case where $\mu _1 = \mu _2 = \mu > 0$ and $L_1 = L_2 = L > \mu$. Theorem 3.1 in [1], on the other hand, also establishes worst-case bounds for some cases of DCA where $\mu _1 \ne \mu _2$, $L_1 \ne L_2$, and where $L_1$ or $L_2$ may not be finite, and $\mu _1$ or $\mu _2$ may be zero. Thus, the new results in this paper do not imply all the known results for DCA from [1] by setting *α = 0*.
5. In Example 1 we showed that the upper bound in Theorem 1 can be tight for specific values of the parameters *μ *, *L*, *α *, and *N*. We conjecture though, that the bound is in fact tight for all values of these parameters that meet the conditions in Theorem 1. In fact, we believe that the optimal value of the SDP problem (4) is precisely the upper bound in Theorem 1 times *L*, when $\mu _1 = \mu _2 = \mu$, and $L_1 = L_2 = L$. This conjecture is based on extensive numerical experiments, as well as a general construction given in the appendix to this paper. This construction was used to generate Example 1, but we believe - although we cannot prove this - that it provides a tight bound for all choices of the parameters. In particular, we conjecture that the general construction in the appendix gives a rank-one optimal solution to the SDP problem (4).

## Data Availability

There are no external data associated with this manuscript. The SDP performance problems we considered in this paper have been implemented in the Matlab software PESTO [19] and the Python version PEPit [11]. Codes allowing the reproduction of the main plots of this work can be found at https://github.com/PerformanceEstimation/Boosted-DCA

## Appendix

Here, we give a general construction that yields Example 1 as the special case where *N=1*, *μ =1*, *L =2*, and *α =1*. After presenting this construction, we will give an indication of how it is derived.

Fix parameters

$$\begin{aligned} 0<\mu<L,\qquad N\in {\mathbb {N}},\qquad 0<\alpha \le \min \{1,2\mu /L\},\qquad \kappa :=\mu /L. \end{aligned}$$

Define

$$\begin{aligned} D:=(1+\kappa \alpha )N+\frac{1}{2(1-\kappa )},\qquad s:=\sqrt{\frac{L}{D}},\qquad \delta :=\frac{s}{L},\qquad q:=1+\kappa \alpha . \end{aligned}$$

Set

$$\begin{aligned} x^1:=0,\qquad x^k:=-(k-1)(1+\alpha )\delta \quad (k=1,\dots ,N+1), \\ y^k:=x^k-\delta ,\qquad z^k:=x^k-\alpha \delta \quad (k=1,\dots ,N), \\ x^\star :=x^{N+1}-\frac{s}{L-\mu }=x^{N+1}-\frac{\delta }{1-\kappa }. \end{aligned}$$

First define endpoint slopes:

$$\begin{aligned} g_2(x^k):=-(k-1)qs\quad (k=1,\dots ,N+1), \qquad g_2(x^\star ):=g_2(x^{N+1})+\mu (x^\star -x^{N+1}). \end{aligned}$$

Define $$g_2:{{\mathbb {R}}}\rightarrow {{\mathbb {R}}}$$ by

$$\begin{aligned} g_2(x):= {\left\{ \begin{array}{ll} g_2(x^\star )+\mu (x-x^\star ), & x\le x^\star ,\\ g_2(x^{N+1})+\mu (x-x^{N+1}), & x^\star \le x\le x^{N+1},\\ g_2(x^{k+1})+L(x-x^{k+1}), & x^{k+1}\le x\le z^k,\ \ k=1,\dots ,N,\\ g_2(x^{k})+\mu (x-x^{k}), & z^k\le x\le x^k,\ \ k=1,\dots ,N,\\ Lx, & x\ge 0. \end{array}\right. } \end{aligned}$$

Now define $f_2$ by integrating $g_2$ from $x^\star$:

$$\begin{aligned} f_2(x):=\int _{x^\star }^{x} g_2(t)\,dt, \quad \text {so that } f_2(x^\star )=0. \end{aligned}$$

Prescribe

$$\begin{aligned} g_1(x^k):=g_2(x^k)+s\quad (k=1,\dots ,N+1), \qquad g_1(y^k):=g_2(x^k)\quad (k=1,\dots ,N). \end{aligned}$$

Define $$g_1:{{\mathbb {R}}}\rightarrow {{\mathbb {R}}}$$ by

$$\begin{aligned} g_1(x):= {\left\{ \begin{array}{ll} g_2(x^\star )+\mu (x-x^\star ), & x\le x^\star ,\\ g_1(x^{N+1})+L(x-x^{N+1}), & x^\star \le x\le x^{N+1},\\ g_1(x^{k+1})+\mu (x-x^{k+1}), & x^{k+1}\le x\le y^k,\ \ k=1,\dots ,N,\\ g_1(y^{k})+L(x-y^{k}), & y^k\le x\le x^k,\ \ k=1,\dots ,N,\\ s+Lx, & x\ge 0. \end{array}\right. } \end{aligned}$$

Now define $f_1$ by integrating $g_1$ from $x^\star$:

$$\begin{aligned} f_1(x):=\int _{x^\star }^{x} g_1(t)\,dt, \quad \text {so that }\, f_1(x^\star )=0. \end{aligned}$$

### Explanation of the construction

The construction above is based on observations of which constraints in the SDP problem (4) are binding at optimality. One may then attempt to construct a solution to these equations. Below we mention the relevant equalities.

Assume $f=f_1-f_2$, $f^\star =0$, $f_1,f_2\in {\mathcal {F}}_{\mu ,L}({\mathbb {R}}^n)$, $0<\mu<L<\infty$, and define

$$\begin{aligned} s_k:= g_1(x^k)-g_2(x^k). \end{aligned}$$

### Algorithmic equalities (for *k=1,… ,N*)

$$\begin{aligned} g_1(y^k)=g_2(x^k), \qquad x^{k+1}=y^k+\alpha \,(y^k-x^k). \end{aligned}$$

### Interpolation equalities used in Table 1 (for *k=1,… ,N*)

$$\begin{aligned} & Q_1(x^k,y^k)=0,\quad Q_1(y^k,x^k)=0,\quad Q_1(x^{k+1},y^k)=0,\quad Q_1(y^k,x^{k+1})=0, \\ & Q_2(x^{k+1},x^k)=0, \end{aligned}$$

where for $$\ell \in \{1,2\}$$,

$$\begin{aligned} \begin{aligned} Q_\ell (u,v):={}&\frac{1}{2\left( 1-\frac{\mu }{L}\right) } \Bigg ( \frac{1}{L}\bigl \Vert g_\ell (u)-g_\ell (v)\bigr \Vert ^2 +\mu \Vert u-v\Vert ^2 -\frac{2\mu }{L}\left\langle g_\ell (v)-g_\ell (u),\,v-u\right\rangle \Bigg ) \\&- f_{\ell }(u)+f_{\ell }(v)+\left\langle g_{\ell }(v),\, u-v\right\rangle . \end{aligned} \end{aligned}$$

### Vanishing squared-norm terms (for *k=1,… ,N*)

$$\begin{aligned} g_1(x^k)-g_2(x^k)-L(x^k-y^k)=0, \\ -g_2(x^k)+g_2(x^{k+1})+(L+\alpha \mu )(x^k-y^k)=0, \\ \begin{aligned} (\alpha \mu -2 (\alpha -1) L)g_1(x^{k+1})&+L(\alpha -2)g_2(x^k)+\alpha (L-\mu )g_2(x^{k+1}) \\&+\alpha L (-\alpha \mu +\mu +L)(x^k-y^k)=0. \end{aligned} \end{aligned}$$

### One-step inequality as equality (for *k=1,… ,N*)

$$\begin{aligned} f(x^{k+1})+\frac{1}{2L}\Vert s_{k+1}\Vert ^2 +\frac{1}{L}\left( \frac{1}{2}+\alpha \frac{\mu }{L}\right) \Vert s_k\Vert ^2 = f(x^k). \end{aligned}$$

### Final descent-lemma equality at $x^{N+1}$

$$\begin{aligned} f(x^{N+1})=\frac{1}{2(L-\mu )}\Vert s_{N+1}\Vert ^2. \end{aligned}$$

### Initial gap

$$\begin{aligned} f(x^1)=1. \end{aligned}$$
